# Environmental occurrence and dissemination of colistin resistance in contaminated aquatic systems: a systematic review of eight observational studies

**DOI:** 10.3389/frabi.2026.1970443

**Published:** 2026-10-07

**Authors:** Alisha Sinha, Jogesh Murmu, Shreya Singh, Aditi Pal, Utkalika Mallick, Poonamrani Mishra, Krushna Chandra Sahoo, Mahesh Chandra Sahu, Sanghamitra Pati

**Affiliations:** 1 ICMR-National Institute of Health Research (NIHR), Bhubaneswar, India; 2 Centre for Biotechnology, Siksha ‘O’ Anusandhan Deemed to beUniversity, Bhubaneswar, Odisha, India; 3 Department of Microbiology, IMS and SUM Hospital, Bhubaneswar, Odisha, India

**Keywords:** antimicrobial resistance, aquatic environments, colistin resistance, environmental surveillance, mcr genes, one health, wastewater

## Abstract

**Introduction:**

Colistin is a last-resort antibiotic used against multidrug-resistant Gram-negative bacterial infections. The emergence of plasmid-mediated colistin resistance, particularly mcr genes, has raised concerns about the environmental persistence and dissemination of resistance determinants. Aquatic environments may serve as reservoirs and dissemination pathways for colistin resistance; however, the available evidence remains fragmented. This systematic review aimed to synthesize evidence on the occurrence, resistance mechanisms, and environmental and potential public health implications of colistin resistance in contaminated aquatic systems.

**Methods:**

A systematic literature search was conducted in five electronic databases up to December 2024 in accordance with PRISMA guidelines. Studies investigating colistin resistance in wastewater, surface water, aquaculture systems, and related aquatic environmental matrices were eligible for inclusion. Following screening and eligibility assessment, eight observational studies were included. Owing to substantial methodological and outcome heterogeneity, a narrative synthesis was performed. Isolate-level phenotypic resistance, sample- or site-level molecular positivity, and metagenomic relative gene abundance were analyzed as separate, non-comparable outcome strata.

**Results:**

A total of 26,740 records were retrieved, of which 20,691 unique records were screened after removal of duplicates. Eight studies met the predefined eligibility criteria. The studies investigated diverse aquatic environments, including slaughterhouse effluents, municipal wastewater, urban rivers, aquaculture systems, and wastewater-associated bioaerosols. Plasmid-mediated mcr genes were identified across several environmental settings and frequently occurred alongside other antimicrobial resistance determinants. Chromosomal mechanisms of colistin resistance were also reported. The reported resistance measures varied substantially between studies, ranging from isolate-level phenotypic resistance and sample/site-level molecular detection to metagenomic relative abundance.

**Discussion:**

The available evidence indicates that contaminated aquatic environments can harbor colistin resistance determinants and may contribute to their environmental dissemination. However, the limited number of eligible studies and substantial methodological heterogeneity prevent direct quantitative comparison or estimation of a common environmental prevalence. Standardized surveillance approaches integrating culture-based methods, molecular detection, and genomic/metagenomic characterization are needed to improve comparability and clarify the environmental pathways of colistin resistance within a One Health framework.

**Systematic review registration:**

https://www.crd.york.ac.uk/PROSPERO/, identifier CRD42024613277.

## 1 Introduction

One of the most important global public health issues of the twenty-first century is antimicrobial resistance (AMR). AMR is believed to undermine the effectiveness of antibiotics that have been the backbone of modern medicine for decades and allow pathogenic microorganisms to survive and thrive that have become resistant to conventional therapeutic approaches (Coque et al., 2023). Among the last resort antimicrobial agents available to control such resistance microbial infections, colistin which is a polymyxin antibiotics that has been revived for use to counteract the rising problem of multidrug resistances, is of particular significance in control of infections caused by carbapenem resistance Enterobacteriaceae and other gram-negative bacteria (Ahmed, 2021). However, one of the last lines of defense against potentially fatal infection has been compromised due to the increased and frequently uncontrolled use of colistin in human medicine, agriculture, veterinary care and aquaculture which has accelerated the emergence and spread of colistin resistance (Memesh et al., 2024).

Resistance to colistin was thought to be chromosomal, with genetic mutations in two-component systems such as *pmrAB*, *phoPQ* or inactivation of *mgrB* all of which affect lipid A moiety modification in bacterial outer membrane (Gogry et al., 2021). Chromosomal resistance typically arises from mutations in the *pmrAB* or *phoPQ* regulatory systems, which can promote the addition of cationic moieties such as phosphoethanolamine to the lipid A component of lipopolysaccharide, thereby reducing the net negative charge of the bacterial outer membrane and decreasing the electrostatic attraction of the positively charged colistin molecule. However, this was revolutionized in 2015 with the identification of the mobilized colistin resistance genes (*mcr*), *mcr-1* in *Escherichia coli* from animals in China harbored on a plasmid (Liu et al., 2016). Since then, ten *mcr* gene variants (*mcr-1* to *mcr-10*) have been found worldwide. These variants are found on a range of plasmid types and are linked to mobile genetic elements like integrons and transposons. This type of resistance can be easily transferred from one species to another in different bacteria due to nature of plasmid, which is involved in horizontal gene transfer which promotes quick spread in environmental, veterinary and clinical settings (Amábile-Cuevas, 2021; Ott and Mellata, 2022).

Additionally, there is proof that aquatic habitats are essential for the preservation and spread of genetic material resistant to colistin. Municipal sewage effluent, hospital wastewater effluent, slaughterhouse effluent, agricultural runoff, and fish farm ponds, where resistant bacteria and resistance genetic material may be found are significant vectors and reservoirs of resistance (Omotoso et al., 2025). These conditions are ideal for the selection and exchange of AMR genetic material because they promote high mixing of various bacterial species along with residual antibiotic residues. Second, inadequately equipped wastewater treatment facilities in developing nations are ineffective at eliminating antibiotic-resistant bacteria or genetic material from effluent that later spreads to surface water systems (Hounmanou et al., 2025).

The problem of the spread of colistin-resistant bacteria and the *mcr* gene in water environments has been recorded in a wide range of locations, from the waters of Asian cities, European slaughterhouse effluent, South American aquaculture, and North American municipal water treatments systems. This pattern suggests that the issue is, in fact, worldwide, which calls for systematic mapping of the prevalence of environmental colistin resistance and its causes (Anyanwu et al., 2020). Significantly, the way this resistance spreads through water has implications that go beyond microbial diversity and ecological health. It may have an impact on human and animal health by enabling resistant organisms to enter the food chain, infect recreational water users, or spread through drinking-water networks (Stec et al., 2022). Airborne transmission of disease through the formation of bioaerosols in water treatment and processing plants provides a further mechanism of potential exposure and complicates the intricate dynamics of transmission. There hasn’t been a concentrated effort to address the problem of colistin resistance in contaminated water resources at the same time that more research is emerging in the field of environmental AMR. The methods used in environmental studies of AMR have varied, as have the results (Anyanwu et al., 2020). There has been a number of studies demonstrating the presence of the *mcr* genes, but the environmental impact of this problem as a burden and genetic determinants of the problem have been a relatively rare exception.

Although previous reviews, including Anyanwu et al. (2020), have summarized the environmental occurrence of antimicrobial resistance and the detection of *mcr* genes, important gaps remain in the systematic synthesis of colistin resistance specifically across contaminated aquatic environments. In particular, previous literature has not comprehensively integrated evidence on isolate-level phenotypic colistin resistance, molecular detection of plasmid-mediated *mcr* variants, chromosomal resistance-associated mutations, and the environmental matrices in which these determinants occur. In addition, differences in detection approaches, including culture-based methods, targeted PCR/qPCR, whole-genome sequencing, and metagenomic approaches, have complicated direct interpretation and comparison of reported findings. The present systematic review therefore focuses specifically on aquatic environments and synthesizes the available evidence on the occurrence of colistin resistance, associated *mcr* variants and chromosomal mechanisms, bacterial hosts, environmental matrices, and relevant resistance-associated characteristics. By distinguishing culture-based evidence of resistant bacteria from culture-independent detection of resistance determinants, this review provides a more focused assessment of the current aquatic-environment evidence base and identifies methodological and geographical gaps that should guide future environmental surveillance.

## 2 Methods

This review was conducted following the Preferred Reporting Items for Systematic Reviews and Meta-Analyses (PRISMA) guidelines (PRISMA statement, 2025). This review registered in PROSPERO (CRD42024613277).

### 2.1 Databases, search strategies, and inclusion criteria

A comprehensive search was conducted across five major electronic databases: PubMed, Embase, Web of Science, Scopus and CINAHL, using a combination of search domains including “colistin,” “water,” and “drug resistance microbial.” This study involved an exhaustive search for all articles up until December 2024, with supplementary literature identified via search engines like Google Scholar and by reviewing the references of relevant literature. Searches were limited to those in English that focused on the topic of colistin resistance in contaminated water bodies (**Supplementary File 1**). The database searches were performed in December 2024, collecting all articles available up until that date, with no minimum publication year. ed on the topic of colistin resistance in contaminated water bodies (**Supplementary File 1**). The database searches were performed in December 2024, collecting all articles available up until that date, with no minimum publication year. The specific search strings used for each database, along with all filters applied (English language restriction and December 2024 cut-off date), can be found in **Supplementary File 1**.

Studies were excluded if water sources were not the primary sample type, colistin resistance was not assessed, relevant outcome data were not available, or studies were available only as an abstract, conference proceeding or without sufficient methodological details. Case reports, case series, editorials, opinion pieces, and narrative reviews were also excluded. Eligible studies included peer-reviewed observational and environmental surveillance studies that investigated colistin resistance in aquatic environments. This included cross-sectional studies, prevalence studies, cohort studies, longitudinal surveillance, and other related observational study designs. All retrieved studies were managed in EndNote for de-duplication and screened via Rayyan in a two-step process including title/abstract screening and full-text screening. A total of 26,740 studies were extracted from five databases, then de-duplication was carried out in one fixed step, removing 4,553 studies in EndNote. Of the 22,187 studies retrieved and imported into Rayyan, 1,496 studies were found to be duplicates and excluded, resulting in 20,691 unique studies imported into Rayyan for title and abstract screening. The PRISMA 2020 flow diagram (**Figure 1**) reports each transition using this reconciled set of figures.

**Figure 1:**
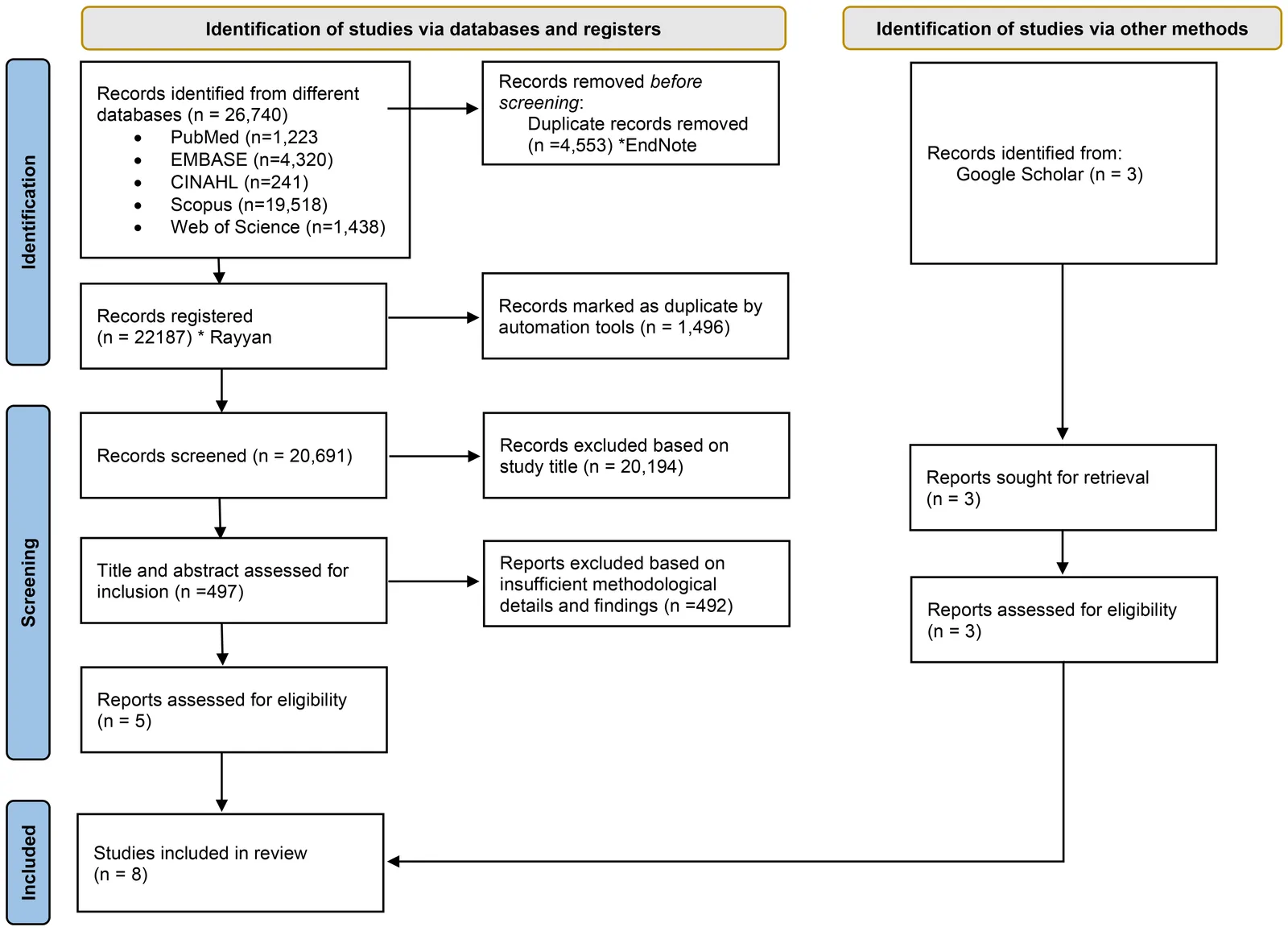
PRISMA flow diagram.

For the purposes of this review, contaminated water systems were defined as aquatic environments affected by anthropogenic activities and potential antimicrobial resistance contamination, including municipal wastewater, hospital wastewater, slaughterhouse effluents, urban rivers and streams receiving sewage inputs, aquaculture systems, wastewater treatment plant influents and effluents, and wastewater-associated environmental matrices. Wastewater-derived bioaerosols and sludge were considered eligible environmental matrices when directly associated with wastewater treatment systems and when studies assessed the occurrence of colistin-resistant bacteria or resistance determinants within these matrices. For studies investigating multiple environmental matrices, data were extracted and synthesized according to the primary aquatic environment or wastewater-associated matrix evaluated by the study.

The search was restricted to English-language publications to ensure consistent assessment of eligibility, methodological quality, and data extraction across the included studies. However, given the global scope of this review, this restriction may have introduced language bias and may have resulted in the omission of relevant evidence published in other languages, particularly from regions where colistin resistance in aquatic environments may be underreported. Therefore, the findings should be interpreted with this limitation in mind.

### 2.2 Data extraction methods

Study selection was performed independently by five reviewers (AS, UM, SS, AP, and PM) using Rayyan, with disagreements resolved through discussion or, when necessary, consultation with two additional reviewers (JM and MS). A copy of the full text article without an extensive abstract was acquired to confirm whether the eligibility criteria had been satisfied. Data extraction was independently duplicated for all eight included studies: each study was extracted in parallel by two reviewers drawn from the five-member extraction team (AS, UM, SS, AP and PM) working from a common piloted extraction form, and the two records for every study were then compared field by field. Extraction was therefore duplicated for the complete set of included studies rather than for a subset. Discrepancies were resolved by returning to the source article and, where agreement was not reached, by adjudication by a third reviewer (MS). Numerical entries in the evidence tables (numerators, denominators, percentages and gene designations) were additionally verified against the source articles by one reviewer (JM) after consensus. Extracted information included study identification details (title, authors, publication year and extraction date), study characteristics (design, study period, country or region, setting, type of water source, and sample size), microbiological findings (bacterial species detected, resistance phenotypes, and specific resistance genes such as *mcr* variants), methodological details (detection methods, molecular typing approaches, and notable innovations), environmental and public health relevance (potential transmission pathways, ecological impacts, and links to human or animal health), and surveillance attributes (type, scope, and duration). Important findings included estimates of prevalence, risk factors, and unique observations. The quality of the studies was assessed using a standard checklist for public health research, covering both observational and experimental studies. All data extracted were compiled into evidence tables to facilitate systematic comparison and synthesis across studies.

### 2.3 Quality assessment of studies

Two reviewers (AS and JM) independently appraised the methodological quality of all included studies using design-specific JBI critical appraisal tools. Prevalence studies (n = 3) were evaluated using the 9-item JBI Prevalence Checklist, which examines the suitability and representativeness of the sample, the validity and consistency of measurement methods, the adequacy of statistical approaches, and potential sources of bias such as sampling and non-response (JBI Critical Appraisal Tools, 2025). Each criterion was scored as “Yes = 1” or “No/Unclear = 0,” and the proportion of applicable criteria met was used to grade the risk of bias as low (≥ 80% of applicable items met), moderate (50–79%) or high (< 50%), such that a higher proportion corresponds to a lower risk of bias and greater methodological quality. For the 9-item prevalence checklist, in which all items were applicable to every study appraised, these thresholds correspond to raw scores of 8–9 (low), 5–7 (moderate) and 0–4 (high). These labels refer to risk of bias rather than to study quality category, and the direction of scoring is applied consistently across all three checklists. Cohort or prospective studies (n = 2) were assessed using the 11-item JBI Cohort Study Checklist (JBI Critical Appraisal Tools, 2025), which evaluates group comparability, exposure measurement, confounding, outcome assessment, and follow-up. The same scoring direction and the same proportional thresholds were applied; because several cohort checklist items were not applicable to environmental surveillance designs, the denominator for these two studies is the number of applicable items rather than the full 11. Cross-sectional studies (n = 3) were reviewed using the 8-item JBI checklist for analytical cross-sectional designs (JBI Critical Appraisal Tools, 2025), focusing on sampling adequacy, measurement validity, identification and management of confounders, and suitability of statistical analyses. Quality ratings followed the same scoring and classification criteria; all eight cross-sectional checklist items were applicable to each of the three studies appraised with this tool, so these thresholds correspond to raw scores of 7–8 (low), 4–6 (moderate) and 0–3 (high) (**Supplementary File 2**). A study-level summary of the total scores, risk-of-bias categories, and the appraisal domains most frequently judged unmet or unclear is presented in **Table 1**, and the complete item-level appraisal is provided in **Supplementary File 2**.

**Table 1:** Summary of JBI critical appraisal (risk-of-bias) ratings for the eight included studies, showing both the applicable-item and full-checklist denominators.

Study (Year)	JBI checklist (total items)	Items met/applicable items (%) [raw score against full checklist]	Risk of bias	Items not applicable to the design; applicable items unmet
Savin et al. (2022)	Prevalence (9)	9/9 (100%) [raw 9/9]	Low	Not applicable: none. Applicable items unmet: none.
Furlan et al. (2021)	Prevalence (9)	4/9 (44%) [raw 4/9]	High	Not applicable: none. Applicable items unmet: appropriate sampling; adequate sample size; standardized measurement of the condition; appropriate statistical analysis; adequate response rate.
Hayashi et al. (2021)	Prevalence (9)	9/9 (100%) [raw 9/9]	Low	Not applicable: none. Applicable items unmet: none.
Isanovic et al. (2024)	Cohort/longitudinal (11)	5/5 (100%) [raw 5/11]	Low (revised from moderate)	Not applicable (6): comparability of two groups; comparable assignment of exposed and unexposed groups; freedom from the outcome at baseline; sufficiency of follow-up time; completeness of follow-up; strategies to address incomplete follow-up. Applicable items unmet: none.
Wang and Smith (2023)	Cohort/longitudinal (11)	4/6 (67%) [raw 4/11]	Moderate (revised from high)	Not applicable (5): comparability of two groups; comparable assignment of exposed and unexposed groups; freedom from the outcome at baseline; completeness of follow-up; strategies to address incomplete follow-up. Applicable items unmet: strategies to deal with confounding factors; appropriate statistical analysis.
Tuo et al. (2018)	Analytical cross-sectional (8)	6/8 (75%) [raw 6/8]	Moderate	Not applicable: none. Applicable items unmet: identification of confounding factors; strategies to deal with confounding factors.
An et al. (2023)	Analytical cross-sectional (8)	6/8 (75%) [raw 6/8]	Moderate	Not applicable: none. Applicable items unmet: identification of confounding factors; strategies to deal with confounding factors.
Nguyen et al. (2021)	Analytical cross-sectional (8)	7/8 (88%) [raw 7/8]	Low	Not applicable: none. Applicable items unmet: strategies to deal with confounding factors.

### 2.4 Data synthesis and analysis

Data from all eligible studies were extracted into standardized evidence tables and synthesized narratively. Substantial heterogeneity in sample types, detection methods and reporting formats precluded the conduct of meta-analysis. Instead, results are compared descriptively across studies, taking care to note differences in water source, geographic context and methodological approach - culture-based, PCR/qPCR or metagenomic techniques. The synthesis also reflected a One Health perspective in examining pathways of environmental contamination, dissemination of *mcr* genes and colistin-resistant organisms and associated public health implications. This allowed the integration of phenotypic, genotypic and environmental data despite the absence of uniformity in reporting required for statistical pooling.

## 3 Results

A total of 26,740 records were retrieved from the database search and imported into Rayyan (n=22,187). After deduplication, 20,691 unique citations remained to undergo title screening. 20,194 records were excluded after title and abstract screening based on predefined eligibility criteria. The remaining 497 reports were sought for retrieval and assessed for eligibility in full text. At this stage, 492 reports were excluded because the studies either lacked sufficient methodological information or focused on unrelated topics, including general water resources, non-relevant contaminants, and/or AMR not associated with water environments; a study-level breakdown of the reasons for full-text exclusion is provided in **Table 2**. The review protocol was registered prospectively in PROSPERO (registration number CRD42024613277). Five studies from the database search met all eligibility criteria, and three further eligible studies were identified through manual searching of Google Scholar and reference lists, giving a total of eight studies that were taken forward for full data extraction and synthesis. The overall screening process, with arithmetically reconciled counts at each stage, is summarized in the PRISMA 2020 flow diagram (**Figure 1**).

**Table 2:** Study-level reasons for exclusion of reports during full-text review (n = 492).

Reason for exclusion at full-text review	Reports excluded, n
Outcome did not assess colistin resistance or *mcr* determinants	168
An aquatic or wastewater environment was not the primary sample type (clinical, animal, or food isolates only)	121
Insufficient methodological detail or full text not retrievable	96
Non-relevant topic (general water quality, other contaminants, or AMR unrelated to water systems)	73
Ineligible publication type (review, conference abstract, editorial, or commentary)	34
**Total excluded at full-text review**	**492**

Bold values indicate the number of reports excluded for each respective reason during full-text review.

**Table 3** shows the main characteristics of the included studies. All included studies adopted observational environmental surveillance designs. Based on their primary methodological approach, three studies were cross-sectional, three were environmental surveillance studies focusing on prevalence, and two were longitudinal/prospective surveillance studies. Because several study characteristics overlapped, these categories are best considered descriptive rather than mutually exclusive classifications (Savin et al., 2022; Furlan et al., 2021; Hayashi et al., 2021; Isanovic et al., 2024; Wang and Smith, 2023; Tuo et al., 2018; An et al., 2023; Nguyen et al., 2021).

**Table 3:** Characteristics of the included studies.

Author (year)	Country/ region	Water source type	Sample size/denominator and study period (as reported in the source study)	Study design	Detection method	Reported resistance outcome/metric (numerator/denominator)	Key findings
Savin et al., 2022	Germany	Slaughterhouse wastewater, municipal WWTPs	185 *Klebsiella* spp. isolates recovered from slaughterhouse wastewater and municipal WWTP samples (denominator for the resistance estimate: 185 isolates). Study period not specified	Descriptive observational environmental surveillance study	Culture-dependent/independent methods, WGS	Isolate-level phenotypic colistin resistance: 32.4% (60/185 *Klebsiella* isolates)	Diverse MDR Klebsiella spp. including high-risk clones; *mcr* genes in livestock wastewater
Furlan et al., 2021	Brazil	Urban stream	1 *E. coli* isolate characterized (February–March 2020). Single-isolate genomic report; no screening denominator reported	Descriptive observational environmental genomic surveillance	WGS, PCR for *mcr* - 1, blaCTX-M-2, blaCMY-2	Isolate-level molecular detection: 1/1 isolate carried *mcr-1.1*. Presented as a raw count; no prevalence can be derived from a single isolate	Co-occurrence of *mcr-1*, blaCTX-M-2, and blaCMY-2 on plasmids and chromosome
Hayashi et al., 2021	Japan	Influent from 4 municipal WWTPs	15 influent samples from 4 municipal WWTPs; 10 colistin- and tigecycline-resistant *Klebsiella* isolates characterized (October–December 2017 and July–September 2018). Total number of *Klebsiella* isolates screened not reported	Environmental surveillance study	PCR, sequencing, and resistance gene mutation analysis	No prevalence estimate reported. Chromosomal resistance-associated *mgrB* and *ramR* disruptions characterized in 10 colistin-resistant *K. pneumoniae* ST29 and *K. quasipneumoniae* isolates from WWTP influents; screening denominator not reported	MgrB and ramR disruptions; *mcr*-negative but chromosomally resistant strains
Isanovic et al., 2024	USA (South Carolina)	Bioaerosols, liquid sludge from 3 WWTPs	Bioaerosol and liquid sludge metagenomes from 3 WWTPs (2019). Culture-independent design; no isolate denominator applicable	Observational longitudinal environmental surveillance study	DNA sequencing + ARG annotation	Metagenomic relative abundance: *mcr* sequences represented 0–0.5% of annotated ARG reads. The denominator is the number of annotated ARG reads per metagenome, not a count of isolates or samples; not comparable with isolate prevalence	*mcr-5* and *mcr-8* most prevalent; airborne spread risk confirmed
Wang and Smith, 2023	USA (California)	County-level wastewater	24 composite wastewater samples from 2 municipal WWTPs (September 2020 – February 2022)	Observational longitudinal environmental surveillance study	Culture + metagenomics + plasmid sequencing	No prevalence estimate reported. *mcr-3.12*, *mcr-4.3*, *mcr-5.1* and *mcr-9.1* identified across the 24 composite samples by multiplex PCR/qPCR, WGS and long-read plasmid sequencing; the number of samples positive for each variant was not reported	Novel *mcr-5* plasmids; *mcr-3.*12 in Aeromonas; *mcr-9.1* in Hafnia
Tuo et al., 2018	China	Funan River (urban river)	1,500 bacterial isolates screened, recovered from 30 Funan River water samples, with a further 10 samples used for ARG profiling (September 2017)	Cross-sectional observational environmental surveillance study	Culture, qPCR, ARG screening	Isolate-level molecular detection: 1.6% (24/1,500 isolates screened) carried *mcr*; *mcr-1* 18/1,500 (1.2%) and *mcr-3* 6/1,500 (0.4%)	High abundance of ARGs near sewage outlets; major ARG pollution source
An et al., 2023	China (Coastal)	Coastal aquaculture zones (27 samples)	27 aquaculture water samples (3 replicates at each of 9 sampling points along >5,000 km of coastline; March–June 2021)	Cross-sectional observational environmental surveillance study	qPCR, conjugation, transformation experiments	Sample-level qPCR detection: *mcr-1* detected in all 27 water samples (27/27; 100% sample positivity), reported as abundance relative to the 16S rRNA gene (4.12 × 10^-^³–4.37 × 10^-^¹) rather than as isolate prevalence. The high-level colistin-resistant isolate characterized (*Ralstonia* pickettii) carried chromosomal rather than *mcr*-mediated resistance	Transmission of colistin resistance mainly vertical; MDR strains widespread
Nguyen et al., 2021	Vietnam	Urban water environments	14 urban water samples (4 urban drainages, 3 rivers, 5 lakes and 2 groundwater sites; February–April 2019)	Cross-sectional observational environmental surveillance study	PCR for *mcr-1*, qPCR, fecal indicator correlation	Sample-level qPCR detection of *mcr-1* across the 14 urban water samples, with the highest concentrations in urban drainages; *mcr-1* abundance correlated with fecal pollution indicators (*E. coli* and crAssphage). [Author query: the figure of 34% sample positivity given in the previous version could not be reconciled with the 14-sample denominator recorded in the data-extraction sheet or with the published report, and has been replaced by the design and outcome as reported; please reinstate a positivity figure only if a verified numerator and denominator are available]	Strong association between *mcr-1* presence and fecal pollution levels

Eight studies met the inclusion criteria, representing diverse geographical settings including Germany (1), Brazil (1), Japan (1), the United States (2), China (2), and Vietnam (1). These studies examined a wide range of contaminated water environments, including slaughterhouse effluents, municipal Wastewater Treatment Plant (WWTP) influents, urban streams and rivers, coastal aquaculture zones, and WWTP bioaerosols and sludge. All the studies had an observational or environmental surveillance design, including cross-sectional (n = 3), prevalence (n = 3; some overlapping), and cohort/longitudinal surveillance studies (n = 2) (**Table 3**).

Some of the studies used culture-based isolation followed by PCR and WGS (Savin et al., 2022; Furlan et al., 2021), and others solely depended on targeted PCR/qPCR methods (An et al., 2023; Nguyen et al., 2021), conjugation or transformation experiments (An et al., 2023), or shotgun metagenomics with ARG annotation (Isanovic et al., 2024; Wang and Smith, 2023). A limited number combined both culture-dependent and culture-independent approaches to capture both hosts and mobile genetic elements that carry resistance genes (**Table 3**).

To account for methodological heterogeneity, phenotypic isolate prevalence and metagenomic relative abundance were analyzed as distinct, non-comparable strata within the synthesis. Quantitative findings are reported according to the outcome unit and denominator used by each study, because the included studies measured colistin resistance in non-comparable ways, namely isolate-level resistance, sample or site positivity, molecular detection of *mcr* variants, and metagenomic relative gene abundance. At the isolate level, Savin et al., 2022 reported colistin resistance in 60 of 185 *Klebsiella* isolates (32.4%) from poultry and slaughterhouse wastewater, whereas Furlan et al., 2021 recovered a single *E. coli* isolate (1/1) carrying *mcr-1.1*; this single-isolate observation is presented as a raw count rather than as a prevalence of 100%. Tuo et al., 2018 screened 1,500 bacterial isolates recovered from 30 Funan River water samples and identified 24 *mcr*-positive isolates (24/1,500; 1.6%), comprising 18 *mcr-1*-positive isolates (18/1,500; 1.2%) and 6 *mcr-3*-positive isolates (6/1,500; 0.4%). In terms of sample and site positivity, An et al., 2023 detected *mcr-1* in all 27 water samples collected as three replicates at each of nine sampling points along more than 5,000 km of coastline (27/27 samples; 100% sample positivity by qPCR), and Nguyen et al., 2021 detected *mcr-1* by qPCR across 14 urban water samples in Hanoi, comprising four urban drainages, three rivers, five lakes and two groundwater sites, with the highest concentrations in urban drainages and with *mcr-1* abundance correlated with fecal pollution indicators. For molecular detection of resistance determinants, Wang and Smith, 2023 identified novel plasmid-borne variants (*mcr-3.12*, *mcr-4.3*, *mcr-5.1* and *mcr-9.1*) across 24 composite wastewater samples, while Hayashi et al., 2021 characterized 10 *mcr*-negative but chromosomally colistin-resistant *Klebsiella* pneumoniae and *K. quasipneumoniae* isolates carrying *mgrB* and *ramR* disruptions, recovered from 15 influent samples across four municipal wastewater treatment plants. Isanovic et al., 2024 reported the relative abundance of *mcr* sequences identified by metagenomic analysis (0-0.5% of annotated ARGs); these sequence-based findings indicate the presence of resistance determinants but cannot by themselves establish viable colistin-resistant organisms or phenotypic resistance. Because these denominators differ and several are very small, percentage estimates derived from single isolates or few samples are not emphasized, and raw numerators and denominators are presented wherever available. **Table 3** reports, for every included study, the unit in which the outcome was measured together with the numerator and the denominator as stated in the source article; where a source article did not report a screening denominator, this is recorded as not reported rather than being converted into a prevalence estimate. Denominators were available for four of the eight studies (Savin et al., 2022; Furlan et al., 2021; Tuo et al., 2018 and An et al., 2023); Hayashi et al., 2021 and Wang and Smith, 2023 reported the number of samples examined but not the number of isolates screened, so no isolate-level prevalence can be derived, Isanovic et al., 2024 reported a relative sequence abundance whose denominator is the number of annotated antimicrobial resistance gene reads rather than a count of isolates or samples, and Nguyen et al., 2021 reported qPCR abundance across 14 water samples rather than a sample positivity proportion (**Table 3**).

The variants of *mcr* were mostly found to be plasmid-mediated, such as *mcr-1*, *mcr-3*, *mcr-4*, *mcr-5*, *mcr-8*, and *mcr-9*. They were often found together with other clinically relevant resistance markers, including bla_CTX-M_ and bla_TEM_. Several mobile genetic elements were involved, such as IncX4 and IncI1 plasmids, small novel plasmids, Tn21-like transposons, and integrons, including intI1. Chromosomal resistance mechanisms, including disruptions in mgrB and ramR, were also identified (Hayashi et al.,2021). The frequency with which each determinant was reported across the included studies is shown in **Figure 2** (**Table 4**).

**Figure 2:**
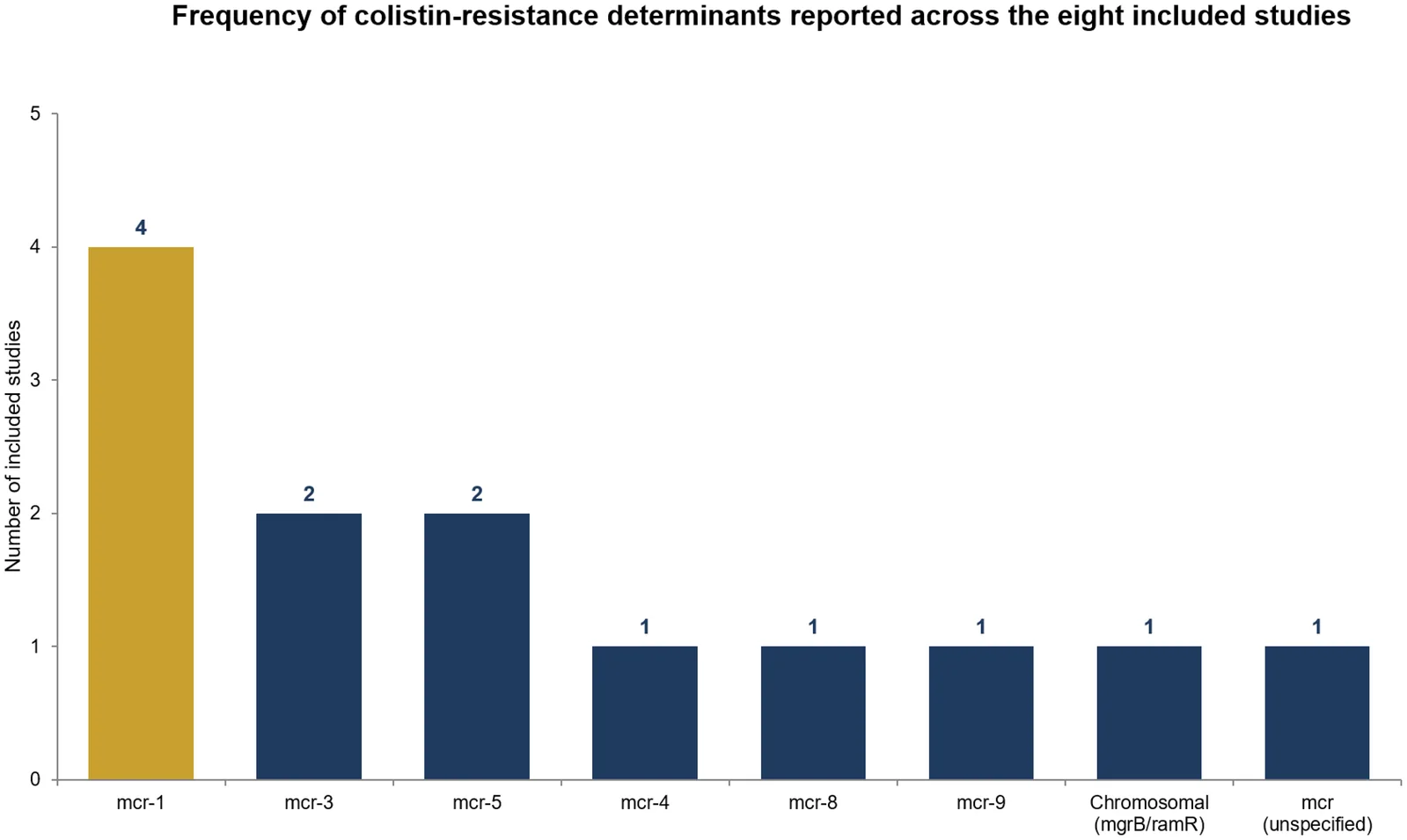
Frequency of colistin-resistance determinants reported across the eight included studies. The unit of counting is the study (n = 8), not the isolate, sample or sequence read. Each study contributes one count to every distinct determinant it reported, so a study reporting several determinants appears in several bars and the bars sum to more than eight (14 study–determinant counts in total). Allelic sub-designations were collapsed to the parent variant (*mcr-1.1* counted as *mcr-1*; *mcr-3.12* as *mcr-3*; *mcr-4.3* as *mcr-4*; *mcr-5.1* as *mcr-5*; *mcr-9.1* as *mcr-9*). Chromosomal resistance is shown as a single category irrespective of the gene involved and comprises two studies (*mgrB*/*ramR* disruption in Hayashi et al., 2021 and chromosomal resistance in the *Ralstonia* pickettii isolate characterized by An et al., 2023, which also contributes a count to *mcr-1* on the basis of qPCR detection in water samples), and studies reporting *mcr* presence without naming a variant are shown as *mcr* (unspecified) rather than being assigned to a named variant (Savin et al., 2022). Isanovic et al., 2024 reported the *mcr-1* to *mcr-10* range in metagenomic data at 0–0.5% relative abundance of annotated antimicrobial resistance gene reads and identified *mcr-5* and *mcr-8* as the predominant variants; that study is therefore counted only for *mcr-5* and *mcr-8*. Had the full reported range been counted instead, one count would have been added to each of the ten named variant categories on the basis of low-abundance sequence reads from a single culture-independent study, and the ranking of variants would have been driven by that study rather than by the evidence as a whole. Gene symbols are shown in italics. Study-level assignments are listed in **Supplementary File 3**, **Table 3**.

**Table 4:** Summary of resistance mechanisms identified.

Author (year)	Resistance genes	Mobile genetic elements	Method of detection	Bacterial species
Savin et al., 2022	Colistin resistance and ESBL determinants reported; specific *mcr* variant not named [Author query: the extraction sheet records ‘ESBL, *mcr-1*’ for this study — please confirm against the source whether *mcr-1* was named, as this determines whether Savin et al. contributes to the *mcr-1* bar or the *mcr* (unspecified) bar in **Figure 2**]	Mobile elements in isolates from pig & poultry wastewater	Culture, PCR, WGS	*K. pneumoniae*, *Klebsiella oxytoca*
Furlan et al., 2021	*mcr-1.1, blaCTX-M-2, blaCMY-2*	IncX4 (*mcr-1.1*), *IncI1-ST12* (*blaCMY-2*), *Tn21-*like	Whole-genome sequencing (WGS), PCR	*Escherichia coli* (ST1775-H137)
Hayashi et al., 2021	Chromosomal resistance (*mgrB, ramR* disruption)	*Tn1548*-like transposon, *IncL/M* plasmids	PCR, sequencing, resistance gene analysis	*K pneumoniae*, *K. quasipneumoniae*
Isanovic et al., 2024	*mcr-1 to mcr-10 (esp. mcr-5, mcr-8)*	Various MGEs on metagenomic contigs	Shotgun metagenomics, ARG annotation	Multiple taxa (not individually listed)
Wang and Smith, 2023	*mcr-3.12, mcr-4.3, mcr-5.1, mcr-9.1*	Novel plasmids (e.g., 8.29 kb, 8.67 kb), transposons	WGS of isolates, metagenomics of plasmids	*Aeromonas hydrophila*, *Hafnia alvei*
Tuo et al., 2018	*mcr-1, mcr-3*	Not specified (but plasmid-mediated assumed)	Culture, PCR, qPCR for ARG abundance	*E. coli*, *Enterobacter cloacae*, *Aeromonas* spp.
An et al., 2023	*mcr-1*, *intI1*, *blaTEM* and other ARGs in water samples; chromosomal colistin resistance in the characterized *Ralstonia* pickettii isolate	MGEs (conjugative plasmids); transformation/conjugation	PCR, conjugation, transformation assays	*Ralstonia pickettii* and other MDRB
Nguyen et al., 2021	*mcr-1*, *intI1*, sul1, tetA, qnrS	Not specified	PCR, qPCR for *mcr-1*, fecal indicator correlation	*E. coli* (used as fecal indicator); environmental community DNA

The major co-resistance patterns, predominant bacterial species, and associated plasmid types identified across the included studies are summarized in **Table 5**. Quality appraisal revealed variable methodological rigor. Scores are expressed as the number of items met out of the number of items applicable to the study design, and risk-of-bias categories were derived from the proportion of applicable items met, as defined in the Methods. Of the three prevalence studies, two met all nine applicable criteria and were rated low risk of bias, whereas the single-isolate genomic report met four of nine applicable criteria and was rated high risk of bias because of limitations in sampling, sample size, standardized measurement and statistical analysis. Of the two cohort or longitudinal surveillance studies, one met all five applicable criteria and was rated low risk of bias and the other met four of six applicable criteria and was rated moderate risk of bias, the unmet items concerning strategies to address confounding and appropriate statistical analysis; six and five items respectively were not applicable to the environmental surveillance design and were excluded from the denominator. Of the three analytical cross-sectional studies, study settings and measurement methods were clearly described in all three, confounding was not adequately addressed in two, which were rated moderate risk of bias, and the remaining study met seven of eight applicable criteria and was rated low risk of bias. Overall, four studies were rated low risk of bias, three moderate and one high (**Table 1**; **Supplementary File 2**).

**Table 5:** Summary of co-resistance patterns, predominant plasmid types, and bacterial species associated with colistin resistance in aquatic environments.

Author (year)	Bacterial species	Colistin resistance determinant	Co-resistance genes	Predominant plasmid/mobile element	Isolates or samples supporting the determinant (n/N, as reported)
Savin et al., 2022	*K. pneumoniae, K. oxytoca*	MCR-associated resistance	ESBL determinants	Mobile genetic elements identified by WGS	60/185 Klebsiella isolates colistin-resistant; mcr presence reported without a variant-level denominator
Furlan et al., 2021	*E. coli* ST1775-H137	*mcr-1.1*	blaCTX-M-2, blaCMY-2	IncX4, IncI1-ST12, Tn21-like transposon	1/1 isolate characterized (single-isolate genomic report)
Hayashi et al., 2021	*K pneumoniae, K. quasipneumoniae*	mgrB and ramR disruption	Multidrug resistance determinants	Tn1548-like transposon, IncL/M plasmids	Chromosomal disruptions characterized in 10 colistin-resistant isolates from 15 influent samples across 4 WWTPs; screening denominator not reported
Isanovic et al., 2024	Multiple taxa	*mcr-1* to *mcr-10*	Multiple ARGs	Diverse mobile genetic elements	0–0.5% relative abundance of annotated ARG reads (metagenomic; no isolate or sample denominator)
Wang and Smith, 2023	*Aeromonas hydrophila, Hafnia alvei*	*mcr-3.12*, *mcr-4.3*, *mcr-5.1*, *mcr-9.1*	Multiple ARGs	Novel plasmids and transposons	Variants identified across 24 composite wastewater samples; per-variant numerator not reported
Tuo et al., 2018	*E. coli, Enterobacter cloacae, Aeromonas* spp.	*mcr-1*, *mcr-3*	Multiple ARGs	Presumed plasmid-mediated	24/1,500 isolates screened (mcr-1 18/1,500; mcr-3 6/1,500)
An et al., 2023	*Ralstonia pickettii* and other MDR bacteria	*mcr-1* (water samples, qPCR); chromosomal resistance in the characterized R. pickettii isolate	blaTEM, intl1 and other ARGs	Conjugative plasmids	mcr-1 detected in 27/27 water samples (9 sampling points × 3 replicates)
Nguyen et al., 2021	Environmental isolates	*mcr-1*	Not specified	Not specified	mcr-1 detected across 14 urban water samples; per-sample numerator not reported

Slaughterhouse effluents and WWTP influents acted as major point sources of multidrug-resistant Enterobacterales (Nguyen et al., 2021). Urban rivers and streams showed enrichment of antimicrobial resistance genes (ARGs) downstream of sewage outlets (Tuo et al., 2018). Coastal aquaculture systems exhibited widespread *mcr-1* contamination, suggesting potential food-chain exposure (An et al., 2023). Isanovic et al., 2024 highlighted possible airborne dissemination through WWTP bioaerosols. Reported impacts included risks of human and animal colonization, environmental spread of novel *mcr* plasmids (Wang and Smith, 2023), and associations between *mcr* presence and fecal pollution indicators (Tuo et al., 2018) (**Table 5**).

These findings suggest that colistin resistance factors are widely spread in different contaminated water sources through both plasmid-mediated and chromosomal ways. However, direct comparisons between these studies are challenging because of significant differences in sampling methods, lab techniques (culture, PCR, or metagenomics), and reporting styles (prevalence at the isolate level or ARG abundance). Many studies also involved very small sample sizes or even single isolates, further reducing the precision of prevalence estimates. While broadening detection, metagenomic studies reporting as relative ARG abundance precluded comparison with the culture-based prevalence estimates directly (**Table 6**).

**Table 6:** Environmental and public health impacts reported.

Author (year)	Reported environmental impact	Evidence of human/animal exposure	Risk pathways identified	Recommended mitigation
Savin et al., 2022	Discharge of MDR *Klebsiella* spp. into surface waters from slaughterhouse wastewater.	Potential colonization of humans and animals via food and water.	Contaminated effluents reaching municipal WWTPs and environment.	Improved slaughterhouse wastewater treatment; One Health surveillance.
Furlan et al., 2021	*mcr*-positive *E. coli* found in polluted urban stream used for recreation.	Direct exposure to MDR *E. coli* in public water.	Water contamination from human and animal waste; plasmid-mediated resistance spread.	Environmental monitoring; control of sewage discharge.
Hayashi et al., 2021	*mcr*-negative but highly resistant *K. pneumoniae* found in WWTP influents.	Presence of human epidemic ST29 clone in wastewater indicates human source.	Human gut bacteria released into WWTP influents; potential spread back into communities.	Monitor WWTP influents; limit further resistance development.
Isanovic et al., 2024	Bioaerosols containing *mcr* genes could spread ARGs kilometers from WWTPs.	Aerosol exposure to surrounding communities.	Airborne transmission via bioaerosols and liquid sludge from WWTPs.	Air quality monitoring near WWTPs; improve containment of bioaerosols.
Wang and Smith, 2023	Novel *mcr* variants and plasmids detected in L.A. wastewater, indicating ARG evolution.	Detection in pathogens like *Aeromonas*, *Hafnia*—potential human infection.	Wastewater-to-environment dissemination of emerging ARGs.	Wastewater surveillance for early ARG detection.
Tuo et al., 2018	High ARG abundance in river water near residential sewage outlets.	Risk through contaminated water for agriculture and human use.	Wastewater runoff, especially near residential discharges, increasing ARG spread.	Restrict untreated sewage discharge; ARG monitoring in urban rivers.
An et al., 2023	MDR and colistin-resistant bacteria widely prevalent in aquaculture farms across >5000 km coastline.	Risk to human consumers and workers in aquaculture.	HGT among aquatic microbes; consumption of contaminated fish/shrimp.	Regulation of antibiotic use in aquaculture; risk assessment of HGT.
Nguyen et al., 2021	Strong correlation of *mcr-1* detection with fecal pollution in Hanoi water sources.	Fecal contamination as indicator of public exposure risk.	Waterborne spread via fecally polluted environments.	Improve sanitation infrastructure; monitor *mcr* - 1 in fecal indicators.

**Figures 2** and **3** provide a visual summary of the core findings. **Figure 2** shows how frequently each colistin-resistance determinant was reported across the eight included studies, and **Figure 3** summarizes the distribution of the included studies by water matrix and by primary detection method. Both figures count studies rather than isolates or samples; the counting rules are defined in the Methods and the study-level assignments underlying every bar are given in **Supplementary File 3**, **Table 1**.

**Figure 3:**
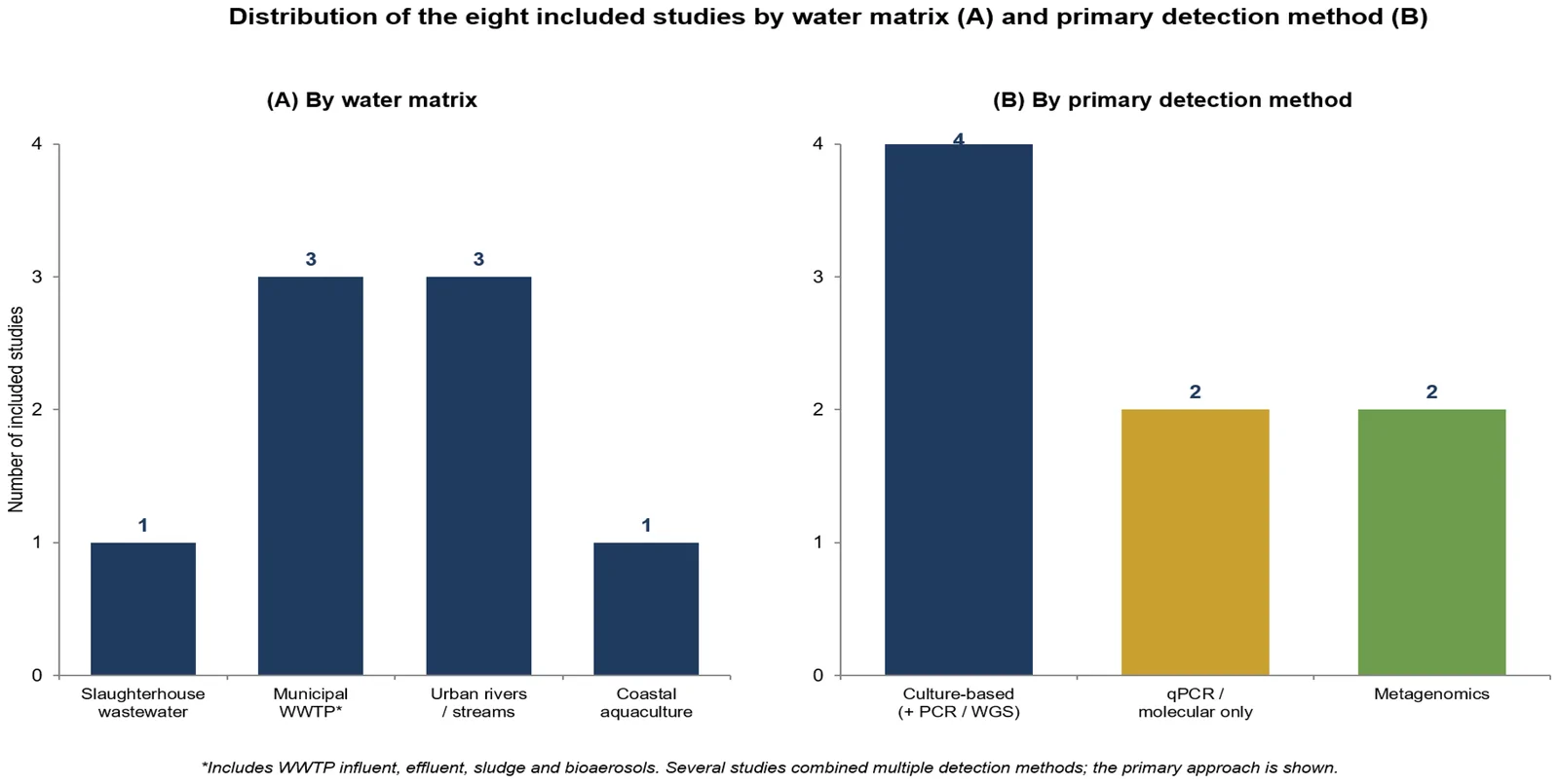
Distribution of the eight included studies by water matrix **(A)** and by primary detection method **(B)**. Each study contributes exactly one count to each panel, so the bars in each panel sum to eight. In panel **(A)** studies sampling more than one matrix were assigned to the single matrix that yielded the samples from which the reported colistin-resistance outcome was derived; on this rule Savin et al., 2022, which sampled both slaughterhouse wastewater and municipal wastewater treatment plants, was assigned to slaughterhouse wastewater because the reported resistance estimate was derived from poultry slaughterhouse wastewater. The municipal wastewater treatment plant category includes influent, effluent, sludge and bioaerosols, and the urban surface water category includes rivers, streams, urban drainages and lakes. In panel **(B)** studies combining several methods were assigned to the single approach that generated the reported outcome, using a fixed order of precedence: culture-based isolation with or without downstream PCR or whole-genome sequencing where the outcome was expressed at isolate level (Savin et al., 2022; Furlan et al., 2021; Hayashi et al., 2021; Tuo et al., 2018); targeted PCR or qPCR where the outcome was derived from environmental nucleic acid without isolation (An et al., 2023; Nguyen et al., 2021); and shotgun metagenomics where the outcome was derived from annotated sequence reads (Isanovic et al., 2024; Wang and Smith, 2023). Wang and Smith, 2023 used both culture and metagenomic approaches and was assigned to metagenomics because the novel plasmid-borne variants it reported were recovered through metagenomic and plasmid sequencing. Study-level assignments are listed in **Supplementary File 3**, **Table 3**.

### 3.1 Descriptive subgroup synthesis

#### 3.1.1 Geographic distribution

The three main geographical areas which featured in the studies include Europe (Germany), Asia (China, Japan, and Vietnam), and the Americas (United States and Brazil). European data were obtained primarily from slaughterhouse waste water and municipal waste water treatment plants where colistin-resistant *Klebsiella* spp. and MDR bacteria were isolated. The Asian literature has described the presence of the mcr gene in urban rivers, waste water treatment plants, and coastal aquaculture farms, which indicates several different environmental sources. Several studies from America have reported on the presence of newly evolved mcr variants and plasmids in both waste water and urban water environments. These patterns of mcr presence according to geographical location, water matrix and detection technique are presented in **Table 7** and shown in **Figure 3**.

**Table 7:** Descriptive subgroup synthesis of included studies according to geographic region, water matrix, and detection method.

Category	Subgroup	Studies	Main findings
Geographic region	Europe	Germany	Colistin-resistant *Klebsiella* spp. in 60/185 isolates (32.4%) from slaughterhouse wastewater; single study
Geographic region	Asia	China, Japan, Vietnam	Widespread *mcr* occurrence in rivers, aquaculture systems, and WWTPs
Geographic region	Americas	USA, Brazil	Novel *mcr* variants and wastewater dissemination
Water matrix	Slaughterhouse wastewater	Savin et al.	Colistin-resistant *Klebsiella* spp. in 60/185 isolates (32.4%); single study
Water matrix	Municipal WWTPs	Hayashi et al., Wang et al., Isanovic et al.	Reservoirs of diverse ARGs and resistance determinants
Water matrix	Urban rivers/streams	Furlan et al., Tuo et al., Nguyen et al.	ARG enrichment associated with sewage contamination
Water matrix	Aquaculture	An et al.	Consistent detection of *mcr* - 1 and MDR bacteria
Water matrix	Bioaerosols/sludge	Isanovic et al.	Potential airborne dissemination pathway
Detection method	Culture + PCR/WGS	Multiple studies	Enabled host and plasmid characterization
Detection method	qPCR	An et al., Nguyen et al., Tuo et al.	Sensitive detection of *mcr* genes
Detection method	Metagenomics	Isanovic et al., Wang et al.	Broad ARG profiling but limited comparability with prevalence estimates

#### 3.1.2 Comparison by water matrix

Colistin resistance was found to occur differently across different aquatic environments. Colistin-resistant Enterobacterales were highly prevalent in slaughterhouse wastewater and represented an important source of resistant bacteria entering municipal wastewater systems. Municipal wastewater treatment plants served as reservoirs of diverse antimicrobial resistance genes and mobile genetic elements. Urban rivers and streams frequently exhibited enrichment of resistance determinants downstream of sewage discharge points. Coastal aquaculture systems demonstrated widespread detection of *mcr-1* and other multidrug resistance determinants, indicating potential food-chain transmission risks. In addition, wastewater-derived bioaerosols emerged as a potential airborne dissemination pathway for colistin resistance genes.

#### 3.1.3 Comparison by detection method

Considerable heterogeneity in methodology was observed among the included studies. Culture-based approaches, when combined with PCR or WGS, enabled characterization of bacterial hosts, resistance determinants, and associated mobile genetic elements. Targeted PCR and qPCR facilitated the detection of specific *mcr* variants in environmental samples. Metagenomic approaches enabled broader profiling of resistance genes and microbial communities; however, detection of *mcr* sequences or ARG reads in environmental DNA indicates the presence of genetic resistance determinants but does not by itself confirm viable resistant bacteria, gene expression, or phenotypic colistin resistance. Therefore, metagenomic and other culture-independent findings should not be directly equated with culture-based measurements of phenotypic resistance, and differences in detection approaches should be considered when interpreting and comparing the reported findings.

Because only eight studies met the eligibility criteria and outcome measures were highly heterogeneous, quantitative subgroup analyses and formal statistical comparisons were not performed. The subgroup synthesis should therefore be interpreted as a descriptive comparison of available evidence.

## 4 Discussion

This systematic review synthesizes the available evidence on the occurrence, genetic determinants, and ecological and public health implications of colistin resistance mediated by plasmid-borne *mcr* genes and chromosomal mutations in aquatic environments. Wastewater, rivers, aquaculture systems, bioaerosols and other environmental waters are now known as an important source of plasmid-mediated *mcr* genes and chromosomal mutations creating colistin resistance. These findings increase a growing body of evidence about the aquatic environment acting as an important reservoir for AMR. This includes last-resort antimicrobials such as colistin. This review suggests there is evidence available to guide future methods of resistance detection, antimicrobial treatment, and policy formulation as the healthcare sector embraces the “One Health framework”.

The presence of *mcr* genes in environmental water bodies across Europe, Asia and the Americas in the eight studies identified in this review indicates that colistin resistance genes occur in the environment across the geographic settings represented here; consistent with wider surveillance reports, this distribution is likely to extend beyond these regions, although such broader inference goes beyond the eight included studies and should be regarded as contextual interpretation (Anyanwu et al., 2020). The presence of colistin-resistant *Klebsiella*, *E. coli*, *Aeromonas*, *Hafnia* and other Enterobacterales species in slaughterhouse wastewater, municipal wastewater and river ecosystems indicates a repeated contamination of these water environments with colistin-resistant bacteria originating from humans, livestock and aquaculture environments (Avatsingh, 2023). The findings corroborate global surveillance studies of *mcr* genes in polluted rivers, influents of WWTPs, aquaculture ponds, and irrigation water.

Slaughterhouse and livestock-related wastewater seem to be a source of particular significance for the spread of resistant organisms. Among the included studies, Savin et al. (2022) reported colistin resistance in 60 of 185 *Klebsiella* isolates (32.4%) recovered from slaughterhouse wastewater. This is consistent with wider evidence that the use of colistin in animal production selects for *mcr*-carrying organisms that may subsequently reach aquatic environments (Anyanwu et al., 2023; Barlaam et al., 2019), although a single study cannot establish the magnitude of that contribution. In the same vein, the detection of *mcr-1* in all 27 coastal aquaculture water samples examined by An et al. (2023) is consistent with reports linking aquaculture to the dissemination of antimicrobial resistance in aquatic environments (Mitchell et al., 2024; Doyle et al., 2025). It should be noted that the high-level colistin-resistant isolate characterized in that study carried chromosomal rather than *mcr*-mediated resistance, so the qPCR detection of *mcr-1* in water and the resistance mechanism of the characterized isolate describe two different observations within the same study.

One of the common findings in studies is that plasmid-borne *mcr* variants, which include *mcr-1*, *mcr-3*, *mcr-5*, *mcr-8*, and *mcr-9*, prevail in causing colistin resistance in aquatic environments. They were frequently found in combination with extended-spectrum β-lactamases (ESBLs) and other multidrug resistance determinants, and the co-selection and co-transmission of *mcr* with ESBL or carbapenemase genes were also reported in many studies (Gāliņa et al., 2021; Liu et al., 2024). Among the most epidemiologically successful vectors of *mcr* across the world are *IncX4, IncI1*, and novel small plasmids, which were identified in studies reviewed. Environmental contaminants such as antibiotic residues, heavy metals, and disinfectants may further promote the persistence and dissemination of *mcr* genes in aquatic environments through co-selection mechanisms (Prasad and Elchuri, 2022). However, such selective factors can often be present in wastewater, agricultural runoff, and aquaculture simultaneously, which means that the presence of multidrug resistance determinants can easily be maintained without direct exposure to colistin. It has been proven through molecular analysis that mcr genes usually reside on mobile plasmids, transposons, and integrons that contain other antibiotic-, metal-, and biocide-resistance genes at the same time. In other words, the presence of just one of those selective factors can contribute to the enrichment of mcr-positive bacteria. Environmental stressors may create conditions conducive to horizontal gene transfer and persistence of resistance determinants; however, the extent to which these processes contribute to *mcr* dissemination in aquatic environments could not be established from the included observational studies. While *de novo* resistance is largely selected within host microbiomes under direct antibiotic pressure, contaminated aquatic systems may act as critical reservoirs and vectors where co-selective agents, such as heavy metals and biocides, may contribute to the persistence and dissemination of pre-existing mobile resistance elements. The high microbial density, nutrient availability, and continuous influx of contaminants in wastewater systems further support genetic exchange and the long-term persistence of resistance elements. These findings suggest that contaminated aquatic environments can serve as reservoirs of colistin resistance and may provide conditions that facilitate the persistence and potential dissemination of *mcr* genes. However, the included studies primarily documented the presence of resistance determinants and mobile genetic elements rather than directly demonstrating genetic recombination, adaptive evolution, or transmission of *mcr* genes within aquatic environments. Across the included studies, colistin resistance was frequently associated with additional clinically important antimicrobial resistance determinants. The most commonly reported co-resistance genes included ESBL-associated genes such as *bla_CTX-M-2_*, bla_CMY-2_, and bla_TEM_, which were often identified together with *mcr* variants on transferable mobile genetic elements. *E. coli* and *Klebsiella* spp. were the predominant bacterial hosts, although resistance determinants were also detected in Aeromonas hydrophila, Hafnia alvei, Enterobacter cloacae, and Ralstonia pickettii. Several epidemiologically important plasmid types, particularly IncX4 and IncI1 plasmids, were identified as major vehicles for *mcr* dissemination. The co-localization of colistin resistance determinants with ESBL and other multidrug resistance genes on the same mobile genetic elements may facilitate co-selection and persistence of resistance under diverse environmental pressures, thereby increasing the risk of dissemination across environmental, animal, and human reservoirs.

Although plasmid-mediated *mcr* genes were predominant, chromosomal resistance mechanisms were also identified. Consistent with **Table 4**, Hayashi et al., 2021 attributed colistin resistance in wastewater-derived *Klebsiella* to chromosomal disruption of *mgrB* and *ramR* (Hayashi et al., 2021). These mutations contribute to stable and high-level resistance and have been reported in clinical strains exposed to sustained antimicrobial pressure, including lineages in which chromosomal and plasmid-mediated determinants occur together (Wang et al., 2023). Such chromosomally resistant clones in WWTP influents (e.g., *K. pneumoniae* ST29) derive contributions to AMR spread in the environment by humans, which is similar to results of wastewater surveillance in Italy, Germany, and the U.S (Triggiano et al., 2020; La Rosa et al., 2025).

### 4.1 Bioaerosols as a potential pathway for environmental dissemination of colistin resistance

One of the findings coming out of this literature review is the possible function of bioaerosols derived from wastewater in the spread of colistin resistance determinants. As reported by Isanovic et al., several variants of the mcr gene were found in bioaerosols and sludge collected from wastewater treatment facilities, including mcr-5 and mcr-8. Wastewater treatment processes such as aeration, mechanical agitation, and sludge handling can generate aerosolized particles containing bacteria and resistance genes.

While bioaerosols present a novel potential transmission vector for colistin-resistance determinants, their broader epidemiological significance has not yet been established beyond the available evidence from Isanovic et al. (2024). Further multi-site environmental validation will be valuable to determine the epidemiological impact of bioaerosol-associated dissemination in comparison with direct waterborne routes.

Although airborne dissemination is increasingly recognized as a dimension of antimicrobial resistance transmission (Jin et al., 2022; Zhou et al., 2021), air sampling is rarely conducted as part of environmental surveillance activities in and around WWTPs. This has resulted in the presence of critical information gaps with respect to AMR genes in environmental bioaerosols. Future research should include monitoring of air, water and sludge in a One Health context. Also, better engineering control systems, procedures for containing aerosols and monitoring occupational exposure can help limit the spread of antimicrobial resistance elements from wastewater treatment plants.

Although the most common type of mechanism found in the articles analyzed is that of plasmid-mediated mcr genes, one must not overlook the role of chromosome-based mechanisms. The disruption of the mgrB and ramR regulatory genes was reported by Hayashi et al., among strains of colistin-resistant *K. pneumoniae* and *K. quasipneumoniae* isolated from municipal waste treatment plants. Unlike mcr-mediated resistance, which can be rapidly disseminated by horizontal gene transfer, chromosomal resistance is mainly propagated through vertical inheritance and clonal expansion. Consequently, resistant bacterial lineages may persist and proliferate in environments subjected to continuous selective pressures, including antibiotic residues, disinfectants, and other environmental contaminants. Wastewater treatment plants may thus serve not only as sinks for the mobile resistant gene pool but also as ecological niche sites where resistant strains may be sustained. Identification of resistant *Klebsiella pneumoniae* ST29 strains indicates the significance of the clonal spread in the environment. As the spread of plasmid-mediated resistance represents a higher threat of rapid spread across different species, chromosomal resistance could be important in ensuring sustained survival of multidrug-resistant bacteria. These results imply that environmental monitoring should target not only mobile genes but also resistant bacterial isolates to gain insights into the mechanism of colistin resistance.

Beyond what was directly measured in the eight included studies, the following human, animal, and ecological health implications are offered as contextual interpretation drawn from the wider literature rather than as direct findings of this review. The widespread detection of colistin-resistant organisms in environmental waters has implications for human, animal, and ecological health: Recreational exposure: Urban rivers and streams used for recreation, such as those in Brazil and Vietnam, contained *mcr*-positive bacteria, raising the risk of colonization or infection (Hassan, 2020). Food chain contamination: Either aquaculture systems or irrigation water may transfer *mcr* genes from aquatic to terrestrial environments, and finally to human contacts via ingestion of contaminated meals (Shen et al., 2019). Drinking-water vulnerability: In regions without adequate water treatment systems, *mcr* genes have been known to migrate to drinking water (Izah and Ogwu, 2025). Environmental selection pressure: The presence of antibiotics, heavy metals, and pesticides in aquatic systems may allow *mcr*-carrying bacteria to persist even in the absence of direct colistin exposure (Snyman et al., 2021). The association between fecal pollution indicators and *mcr-1* abundance reported by Nguyen et al. (2021), one of the included studies, indicates that human fecal contamination is a plausible source of *mcr-1* in urban water environments; comparable carriage across humans, animals and the farm environment has been described elsewhere in Vietnam (Nguyen et al., 2022).

As described in this review, significant heterogeneity in strategies for sampling, sequencing, detection, and metrics of reporting hindered cross-study comparisons. This finding follows concerns from previous environmental AMR synopses regarding the need for standardized infection control practices, harmonized surveillance protocols, and uniform molecular methodologies in environmental surveillance (Struelens et al., 2024; Liguori et al., 2022). Single isolates and small sample sizes further limit generalizability. Culture-based methods tended to underreport *mcr* relative abundance when compared to metagenomic approaches, while metagenomic approaches lacked isolate-level context. Hence, both approaches must be utilized in order to obtain robust pan-environmental surveillance. These limitations also bear on the confidence that can be placed in individual conclusions. The risk-of-bias appraisal (**Table 1**) rated four studies as low risk, three as moderate risk and one as high risk when scored against the items applicable to each design. Confidence is lowest for the single-isolate genomic report, which was rated high risk of bias and from which no prevalence can be derived, and for the one longitudinal surveillance study rated moderate risk on account of unaddressed confounding and limited statistical analysis. Conclusions are therefore weighted toward the better-quality evidence, and the prevalence figures derived from very small denominators should be read as indicative rather than precise.

Despite the worldwide implications of colistin resistance, the eight included studies were geographically concentrated in Germany, Brazil, Japan, the USA, China, and Vietnam, and no eligible study from India was identified. Therefore, the absence of India-specific evidence should be interpreted as a potential surveillance gap rather than evidence for the absence of environmental colistin resistance. Information from India and other low- and middle-income countries (LMICs), including agricultural practices, wastewater management, and sanitation challenges, is presented here only as contextual information that may be relevant to future environmental surveillance. These contextual considerations were not directly evaluated by the included studies and therefore cannot be considered findings of this systematic review. Future studies in underrepresented regions, including India and other LMICs, are warranted to determine the occurrence, distribution, and molecular characteristics of colistin resistance in aquatic environments.

## 5 Limitations

This review relied heavily on a varied set of results which had large differences in the types of samples, the number of samples analyzed, the tools used in obtaining resistance data (culture-based methods, qPCR, metagenomics), as well as the representation of results. This reduced the ability to collate results in a combined manner, making it difficult to compare results. Some studies also had small sample sizes or results dependent on isolated cases, which reduced the accuracy of results. This value, however, represents one of the earliest systematic reviews undertaken involving the effects of colistin resistance in environmental water bodies, incorporating phenotypic results together with genomic findings, which revealed the principal human determinants promoting resistance. Although more than 22,000 records were initially identified, only eight studies fulfilled all predefined eligibility criteria. The high exclusion rate during initial screening (>99%) reflects the broad Boolean string combinations used across major databases, which captured general antimicrobial-resistance literature that lacked specific aquatic colistin endpoints. The limited number of eligible studies may reflect gaps in the available literature, although the evidence base remains limited and should be interpreted cautiously. Many environmental antimicrobial resistance studies focus on overall resistance patterns without specifically investigating colistin resistance or *mcr* genes in aquatic systems. Furthermore, substantial heterogeneity in environmental matrices, laboratory methodologies, molecular detection techniques, and reporting standards reduced the number of studies eligible for inclusion. The absence of studies suggests the need for standardized protocols for environmental surveillance and increased research efforts, particularly in low- and middle-income countries where challenges in wastewater management and antimicrobial use may play a substantial role in the environmental dissemination of colistin resistance. The review was restricted to English-language publications, which may have introduced language bias. The predominantly cross-sectional studies could not establish the directionality of colistin-resistance transmission. Differences in the analytical sensitivity of culture-based, qPCR, and metagenomic methods may also affect detection and cross-study comparisons. Furthermore, environmental DNA-based methods cannot confirm the viability of organisms carrying resistance genes.

## 6 Impact of the study and future planning

This review further adds to an increasing amount of global evidence that has now made it quite evident that resistance to colistin has emerged beyond hospitals and farming systems and has now entered environmental water systems as well. It is quite worrisome to realize that colistin remains to be one of the last available resources against MDR Gram-Negative bacterial infections. The recurrence of *mcr* gene copies along with ESBL characteristics further raises concerns of what could be termed as “co-selection,” such that any other antibiotic or even any other chemical could unintentionally contribute to maintaining or even increasing colistin resistance in aquatic environments. The included studies were drawn from six countries in Europe, Asia and the Americas, and none of the major producers of aquaculture and poultry in South Asia was represented. This uneven geographical coverage is a disparity in environmental surveillance effort rather than a finding about where colistin resistance occurs, and it defines where additional primary evidence is most needed.

The practical implications follow from that surveillance disparity rather than from any country-specific finding of this review. Regions that combine intensive animal and aquaculture production with incomplete wastewater treatment are the settings in which environmental colistin resistance is least well characterized and most plausibly consequential, and they are also the settings least represented in the eight included studies. India is one such example: no eligible Indian study was identified here, although national reports of *mcr* determinants in human, veterinary and environmental isolates exist outside the scope of this review. Priorities for these settings are the integration of environmental sampling into existing antimicrobial resistance surveillance, the harmonization of sampling frames and molecular methods so that results are comparable between sites, and the reporting of outcomes with numerators and denominators. These are contextual considerations drawn from the wider literature and from the geographical distribution of the included studies; they were not evaluated by the included studies and are not findings of this systematic review.

## 7 Conclusion

This systematic review highlights the occurrence of colistin resistance across different aquatic matrices, including the detection of plasmid-borne *mcr* variants and chromosomal resistance-associated mutations among the included studies. These findings indicate that aquatic environments may serve as important reservoirs of colistin resistance and may contribute to its persistence and dissemination. However, the current evidence base remains small, heterogeneous, and fragmented, with substantial variation in sampling approaches, aquatic matrices, resistance assessment, and molecular characterization across the eight included studies. Therefore, the available evidence does not yet permit a comprehensive assessment of the extent or transmission dynamics of colistin resistance in aquatic environments. Future studies should prioritize standardized environmental surveillance, molecular characterization of *mcr* variants and chromosomal resistance mechanisms, and integrated genomic approaches to better characterize the occurrence and dissemination of colistin resistance. Strengthening wastewater management and incorporating environmental reservoirs into One Health antimicrobial-resistance surveillance frameworks may further support coordinated efforts to limit the environmental persistence and dissemination of colistin resistance.

## Data Availability

The original contributions presented in the study are included in the article/**Supplementary Material**. Further inquiries can be directed to the corresponding author.

## Supplementary material

Supplementary File 1 Complete database-specific search strategy, including the full Boolean search strings, field tags, applied filters and database-specific record yields for PubMed, Embase, Web of Science, Scopus and CINAHL, reconciled with the counts reported in the Methods, the Results and **Figure 1**.

Supplementary File 2 Item-level JBI critical appraisal (risk-of-bias) table for all eight included studies, showing for every item whether it was met, unmet or not applicable to the study design, together with the applicable-item and full-checklist scores and the derived risk-of-bias category (summarized in **Table 1**).

Supplementary File 3 The study-level assignments underlying **Figure 2** and **3**.
