# Supplementary material for: Environmental occurrence and dissemination of colistin resistance in contaminated aquatic systems: a systematic review of eight observational studies
Source: Front Antibiot. 2026 Oct 7;5:1970443. doi: 10.3389/frabi.2026.1970443 (PMC13683747; doi:10.3389/frabi.2026.1970443)
Supplement: Supplementary file 1 [file Table1.docx]

**Search Strategy**

| **Sl. No** | **Database** | **Search Strategy** | **Results** |
| --- | --- | --- | --- |
| 1 | PubMed | ("colistin"[MeSH Terms] OR "colistin"[All Fields] OR "colistine"[All Fields] OR "colistin*"[All Fields]) OR "polymyxin e"[All Fields]) OR ("colistin sulphate"[All Fields] AND "sulfate"[All Fields]) OR "colistin sulfate"[All Fields]) OR "sulfate colistin"[All Fields]) AND "contaminant"[All Fields] OR "contaminant*"[All Fields] OR "contaminating"[All Fields] OR "contamination*"[All Fields] OR "contaminative"[All Fields] OR "contamined"[All Fields] OR "water"[MeSH Terms] OR "water*"[All Fields] OR "source"[All Fields] OR "source*"[All Fields] OR "wastewater"[MeSH Terms] OR "waste water"[All Fields] OR “pathogen” [All Fields] OR “waterborne” [All Fields] AND "drug resistance, microbial"[MeSH Terms] OR "microbial drug resistance"[All Fields] OR ("drug"[All Fields] AND "resistance"[All Fields] AND "microbial"[All Fields]) OR "antibiotic resistance"[All Fields] OR ("antibiotic"[All Fields] AND "resistance"[All Fields] AND "microbial"[All Fields]) OR ("drug"[All Fields] AND "resistances"[All Fields] AND "microbial"[All Fields]) OR "antibiotic resistance microbial"[All Fields] OR "antimicrobial drug resistance"[All Fields] OR ("antimicrobial"[All Fields] AND "drug resistance"[All Fields]) OR ("antimicrobial"[All Fields] AND "drug resistances"[All Fields]) OR "antimicrobial resistance drug"[All Fields] OR "drug antimicrobial resistance"[All Fields] | 1,223 |
| 2 | Embase | (colistin/exp OR colistin OR colistine OR colistin*) OR 'polymyxin e' OR ('colistin sulphate' AND sulfate) OR 'colistin sulfate' OR 'sulfate colistin' AND contaminant OR contaminant* OR contaminating OR contamination* OR contaminative OR contamined OR water/exp OR water* OR source OR source* OR wastewater/exp OR 'waste water' OR pathogen OR 'water borne disease' AND 'drug resistance, microbial'/exp OR 'microbial drug resistance' OR (drug AND resistance AND microbial) OR 'antibiotic resistance' OR (antibiotic AND resistance AND microbial) OR (drug AND resistances AND microbial) OR 'antibiotic resistance microbial' OR 'antimicrobial drug resistance' OR (antimicrobial AND 'drug resistance') OR (antimicrobial AND 'drug resistances') OR 'antimicrobial resistance drug' OR 'drug antimicrobial resistance' | 4320 |
| 3 | CINAHL | ((MH colistin+) OR colistin OR colistine OR colistin*) OR "polymyxin e"OR ("colistin sulphate" AND sulfate) OR "colistin sulfate" OR "sulfate colistin" AND contaminant OR contaminant* OR contaminating OR contamination* OR contaminative OR contamined OR water/exp OR water* OR source OR source* OR wastewater/exp OR 'waste water' OR pathogen OR waterborne AND 'drug resistance, microbial'/exp OR 'microbial drug resistance' OR (drug AND resistance AND microbial) OR 'antibiotic resistance' OR (antibiotic AND resistance AND microbial) OR (drug AND resistances AND microbial) OR 'antibiotic resistance microbial' OR 'antimicrobial drug resistance' OR (antimicrobial AND 'drug resistance') OR (antimicrobial AND 'drug resistances') OR 'antimicrobial resistance drug' OR 'drug antimicrobial resistance' | 241 |
| 4 | Scopus | ( INDEXTERMS ( colistin ) OR ALL ( colistin ) OR ALL ( colistine ) OR ALL ( colistin* ) ) OR ALL ( "polymyxin e" ) OR ( ALL ( "colistin sulphate" ) AND ALL ( sulfate ) ) OR ALL ( "colistin sulfate" ) OR ALL ( "sulfate colistin" ) AND ALL ( contaminant ) OR ALL ( contaminant* ) OR ALL ( contaminating ) OR ALL ( contamination* ) OR ALL ( contaminative ) OR ALL ( contamined ) OR INDEXTERMS ( water ) OR ALL ( water* ) OR ALL ( source ) OR ALL ( source* ) OR INDEXTERMS ( wastewater ) OR ALL ( "waste water" ) OR ALL ( pathogen ) OR ALL ( waterborne ) AND TITLE-ABS-KEY ( "drug resistance, microbial" OR "microbial drug resistance" OR ( drug AND resistance AND microbial ) OR "antibiotic resistance" OR ( antibiotic AND resistance AND microbial ) OR ( drug AND resistances AND microbial ) OR "antibiotic resistance microbial" OR "antimicrobial drug resistance" OR ( antimicrobial AND "drug resistance" ) OR ( antimicrobial AND "drug resistances" ) OR "antimicrobial resistance drug" OR "drug antimicrobial resistance" ) | 19518 |
| 5 | Web of Science | (colistin OR colistin OR colistin OR colistin*) OR "polymyxin e"OR ("colistin sulphate" AND sulfate) OR "colistin sulfate" OR "sulfate colistin" AND contaminant OR contaminant* OR contaminating OR contamination* OR contamination OR contained OR water OR water* OR source OR source* OR wastewater OR "waste water" OR pathogen OR waterborne AND "drug resistance, microbial" OR "microbial drug resistance" OR (drug AND resistance AND microbial) OR "antibiotic resistance" OR (antibiotic AND resistance AND microbial) OR (drug AND resistances AND microbial) OR "antibiotic resistance microbial" OR "antimicrobial drug resistance" OR (antimicrobial AND "drug resistance") OR (antimicrobial AND "drug resistances") OR "antimicrobial resistance drug" OR "drug antimicrobial resistance" | 1,438 |
