# Supplementary material for: Environmental occurrence and dissemination of colistin resistance in contaminated aquatic systems: a systematic review of eight observational studies
Source: Front Antibiot. 2026 Oct 7;5:1970443. doi: 10.3389/frabi.2026.1970443 (PMC13683747; doi:10.3389/frabi.2026.1970443)
Supplement: Supplementary file 2 [file Table2.docx]

**Supplementary File 2. Item-level JBI critical appraisal (risk of bias) of the included studies**

Each item was scored as Yes = 1 or No/Unclear = 0. Items that are structurally inapplicable to a given study design are marked NA and are excluded from the denominator; an item was designated NA only where the design made the item impossible to satisfy, and not where the item was applicable but unmet or unclear, which continued to be scored 0. Applicability was judged independently and in duplicate by two appraisers (AS and JM), with disagreements resolved by discussion. Each study was appraised with the single checklist matching its design; the two checklists that do not apply to a study are marked NA throughout. The appraisal score is expressed as the number of items met out of the number of applicable items, and the risk-of-bias category is derived from the proportion of applicable items met, using one set of thresholds for all three checklists: low (≥ 80% of applicable items met), moderate (50–79%) and high (< 50%). A higher proportion therefore corresponds to a lower risk of bias.

| **JBI Prevalence Checklist** | Savin et al., 2022 | Furlan et al., 2021 | Hayashi et al., 2021 | Isanovic et al., 2024 | Wang and Smith, 2023 | Tuo et al., 2018 | An et al., 2023 | Nguyen et al., 2021 |
| --- | --- | --- | --- | --- | --- | --- | --- | --- |
| Was the sample frame appropriate to address the target population? | Yes | Yes | Yes | NA | NA | NA | NA | NA |
| Were study participants sampled in an appropriate way? | Yes | No | Yes | NA | NA | NA | NA | NA |
| Was the sample size adequate? | Yes | No | Yes | NA | NA | NA | NA | NA |
| Were the study subjects and the setting described in detail? | Yes | Yes | Yes | NA | NA | NA | NA | NA |
| Was the data analysis conducted with sufficient coverage of the identified sample? | Yes | Yes | Yes | NA | NA | NA | NA | NA |
| Were valid methods used for the identification of the condition? | Yes | Yes | Yes | NA | NA | NA | NA | NA |
| Was the condition measured in a standard, reliable way for all participants? | Yes | No | Yes | NA | NA | NA | NA | NA |
| Was there appropriate statistical analysis? | Yes | No | Yes | NA | NA | NA | NA | NA |
| Was the response rate adequate, and if not, was the low response rate managed appropriately? | Yes | No | Yes | NA | NA | NA | NA | NA |
| **Overall Risk of Bias** | **Low Risk** | **High Risk** | **Low Risk** | **NA** | **NA** | **NA** | **NA** | **NA** |
| **JBI Checklist for Cohort Studies** |  |  |  |  |  |  |  |  |
| Were the two groups similar and recruited from the same population? | NA | NA | NA | NA | NA | NA | NA | NA |
| Were the exposures measured similarly to assign people to both exposed and unexposed groups? | NA | NA | NA | NA | NA | NA | NA | NA |
| Was the exposure measured in a valid and reliable way? | NA | NA | NA | Yes | Yes | NA | NA | NA |
| Were confounding factors identified? | NA | NA | NA | Yes | Yes | NA | NA | NA |
| Were strategies to deal with confounding factors stated? | NA | NA | NA | Yes | No | NA | NA | NA |
| Were the groups/participants free of the outcome at the start of the study (or at the moment of exposure)? | NA | NA | NA | NA | NA | NA | NA | NA |
| Were the outcomes measured in a valid and reliable way? | NA | NA | NA | Yes | Yes | NA | NA | NA |
| Was the follow up time reported and sufficient to be long enough for outcomes to occur? | NA | NA | NA | NA | Yes | NA | NA | NA |
| Was follow up complete, and if not, were the reasons to loss to follow up described and explored? | NA | NA | NA | NA | NA | NA | NA | NA |
| Were strategies to address incomplete follow up utilized? | NA | NA | NA | NA | NA | NA | NA | NA |
| Was appropriate statistical analysis used? | NA | NA | NA | Yes | No | NA | NA | NA |
| **Overall Risk of Bias** | **NA** | **NA** | **NA** | **Low Risk** | **Moderate Risk** | **NA** | **NA** | **NA** |
| **JBI Checklist for Analytical Cross-sectional Studies** |  |  |  |  |  |  |  |  |
| Were the criteria for inclusion in the sample clearly defined? | NA | NA | NA | NA | NA | Yes | Yes | Yes |
| Were the study subjects and the setting described in detail? | NA | NA | NA | NA | NA | Yes | Yes | Yes |
| Was the exposure measured in a valid and reliable way? | NA | NA | NA | NA | NA | Yes | Yes | Yes |
| Were objective, standard criteria used for measurement of the condition? | NA | NA | NA | NA | NA | Yes | Yes | Yes |
| Were confounding factors identified? | NA | NA | NA | NA | NA | No | No | Yes |
| Were strategies to deal with confounding factors stated? | NA | NA | NA | NA | NA | No | No | No |
| Were the outcomes measured in a valid and reliable way? | NA | NA | NA | NA | NA | Yes | Yes | Yes |
| Was appropriate statistical analysis used? | NA | NA | NA | NA | NA | Yes | Yes | Yes |
| **Overall Risk of Bias** | **NA** | **NA** | **NA** | **NA** | **NA** | **Moderate Risk** | **Moderate Risk** | **Low Risk** |
| **Summary across checklists** | **Savin et al., 2022** | **Furlan et al., 2021** | **Hayashi et al., 2021** | **Isanovic et al., 2024** | **Wang and Smith, 2023** | **Tuo et al., 2018** | **An et al., 2023** | **Nguyen et al., 2021** |
| **Checklist applied** | Prevalence (9) | Prevalence (9) | Prevalence (9) | Cohort / longitudinal (11) | Cohort / longitudinal (11) | Cross-sectional (8) | Cross-sectional (8) | Cross-sectional (8) |
| **Items applicable to the design (n)** | 9 | 9 | 9 | 5 | 6 | 8 | 8 | 8 |
| **Items not applicable (n)** | 0 | 0 | 0 | 6 | 5 | 0 | 0 | 0 |
| **Items met (n)** | 9 | 4 | 9 | 5 | 4 | 6 | 6 | 7 |
| **Items met / applicable (%)** | 9/9 (100%) | 4/9 (44%) | 9/9 (100%) | 5/5 (100%) | 4/6 (67%) | 6/8 (75%) | 6/8 (75%) | 7/8 (88%) |
| **Raw score against full checklist** | 9/9 | 4/9 | 9/9 | 5/11 | 4/11 | 6/8 | 6/8 | 7/8 |
| **Risk of bias (from % of applicable items met)** | **Low** | **High** | **Low** | **Low** | **Moderate** | **Moderate** | **Moderate** | **Low** |
