# Supplementary material for: Environmental occurrence and dissemination of colistin resistance in contaminated aquatic systems: a systematic review of eight observational studies
Source: Front Antibiot. 2026 Oct 7;5:1970443. doi: 10.3389/frabi.2026.1970443 (PMC13683747; doi:10.3389/frabi.2026.1970443)
Supplement: Supplementary file 3 [file Table3.docx]

**Supplementary table 3:** Study-level counting assignments used to construct Figures 2 and 3

| **Study (Year)** | **Determinants counted in Figure 2** | **Counting note** | **Figure 3A: water matrix** | **Figure 3B: primary detection method** |
| --- | --- | --- | --- | --- |
| Savin et al. (2022) | mcr (unspecified) [1 count] | Colistin resistance and ESBL determinants reported without a named mcr variant; assigned to the mcr (unspecified) category rather than to mcr-1 | Slaughterhouse wastewater (municipal WWTPs also sampled; assigned to the matrix yielding the reported resistance estimate) | Culture-based (± PCR/WGS); outcome expressed at isolate level |
| Furlan et al. (2021) | mcr-1 [1 count] | mcr-1.1 collapsed to the parent variant mcr-1 | Urban surface water (urban stream) | Culture-based (± PCR/WGS); outcome expressed at isolate level |
| Hayashi et al. (2021) | Chromosomal resistance [1 count] | No mcr determinant reported; mgrB and ramR disruption counted once in the chromosomal category | Municipal WWTP (influent) | Culture-based (± PCR/WGS); outcome expressed at isolate level |
| Isanovic et al. (2024) | mcr-5; mcr-8 [2 counts] | mcr-1 to mcr-10 reported at 0–0.5% relative abundance of annotated ARG reads; counted only for the two variants identified as predominant, so that low-abundance reads from one culture-independent study do not populate all ten variant categories | Municipal WWTP (bioaerosols and liquid sludge) | Metagenomics; outcome derived from annotated sequence reads |
| Wang and Smith (2023) | mcr-3; mcr-4; mcr-5; mcr-9 [4 counts] | mcr-3.12, mcr-4.3, mcr-5.1 and mcr-9.1 collapsed to their parent variants | Municipal WWTP (county wastewater) | Metagenomics; culture was also used, but the reported plasmid-borne variants were recovered through metagenomic and plasmid sequencing |
| Tuo et al. (2018) | mcr-1; mcr-3 [2 counts] | Two determinants reported among 24 mcr-positive isolates | Urban surface water (urban river) | Culture-based (± PCR/WGS); outcome expressed at isolate level |
| An et al. (2023) | mcr-1; chromosomal resistance [2 counts] | mcr-1 detected in water samples by qPCR; chromosomal resistance in the characterised Ralstonia pickettii isolate counted once in the chromosomal category | Coastal aquaculture | Targeted PCR/qPCR; outcome derived from environmental nucleic acid without isolation |
| Nguyen et al. (2021) | mcr-1 [1 count] | Single determinant reported | Urban surface water (urban drainages, rivers, lakes and groundwater) | Targeted PCR/qPCR; outcome derived from environmental nucleic acid without isolation |
| **Total** | **14 study–determinant counts across 8 studies** | **Bars in Figure 2 sum to more than 8 because a study may report several determinants** | **8 studies, one count each (1 + 3 + 3 + 1)** | **8 studies, one count each (4 + 2 + 2)** |

**Note:** The unit of counting in both figures is the study (n = 8), not the isolate, sample or sequence read. In Figure 2 a study contributes one count to every distinct determinant it reported, so the bars sum to 14 rather than 8. In Figure 3 each study contributes exactly one count to each panel, so the bars in each panel sum to 8; where a study sampled several matrices or applied several detection methods, it was assigned to the single matrix or method that generated the reported colistin-resistance outcome, following the rules set out in the Methods. This table is provided so that every bar in Figures 2 and 3 can be traced to the individual studies that generated it.
